# Hereditary hemorrhagic telangiectasia with hepatic arteriovenous shunt diagnosed due to liver damage

**DOI:** 10.1007/s12328-024-01923-0

**Published:** 2024-03-04

**Authors:** Satoru Hagiwara, Toru Takase, Itsuki Oda, Yoriaki Komeda, Naoshi Nishida, Akihiro Yoshida, Tomoki Yamamoto, Takuya Matsubara, Masatoshi Kudo

**Affiliations:** 1 Department of Gastroenterology and Hepatology, Kindai University Faculty of Medicine, 377-2 Ohno-Higashi, Osaka-Sayama, Osaka 589-8511 Japan; 2 Department of Cardiovascular Medicine, Kindai University Faculty of Medicine, Osaka, Japan; 3 Department of Clinical Genetics, Kindai University Faculty of Medicine, Osaka, Japan

**Keywords:** Hereditary hemorrhagic telangiectasia, Hepatic arteriovenous shunt, Liver damage

## Abstract

A 53-year-old woman was diagnosed with liver dysfunction in August 20XX. Computed tomography (CT) revealed multiple hepatic AV shunts, and she was placed under observation. In March 20XX + 3, she developed back pain, and CT performed during an emergency hospital visit showed evidence of intrahepatic bile duct dilatation. She was referred to our gastroenterology department in May 20XX + 3. We conducted investigations on suspicion of hereditary hemorrhagic telangiectasia (HHT) with hepatic AV shunting based on contrast-enhanced CT performed at another hospital. HHT is generally discovered due to epistaxis, but there are also cases where it is diagnosed during examination of liver damage.

## Introduction

Hereditary hemorrhagic telangiectasia (HHT), also known as Osler-Rendu-Weber disease [1, 2], is a systemic vascular disease with four pathognomonic clinical features, viz. recurrent epistaxis, peripheral vasodilation of the skin and mucosa, visceral lesions [arteriovenous malformations (AVMs)], and autosomal dominant inheritance [3, 4]. HHT is broadly classified into two types based on the causative gene: *ENG* in HHT1, and *ACVRL-1* in HHT2 [5–7]. Pulmonary hypertension is an important complication of HHT [8, 9]. Pulmonary hypertension is broadly divided into pulmonary arterial hypertension, which causes stenosis due to remodeling of pulmonary blood vessels, and pulmonary hypertension, which occurs due to increased cardiac output due to arteriovenous shunts. However, some cases manifest as a mixture of both of these patterns [10]. Herein, we report a case of Osler-Rendu-Weber disease with a hepatic AV shunt complicated by pulmonary arterial hypertension, which was diagnosed as a result of liver damage.

## Case report

The patient was a 53-year-old woman who presented with a chief complaint of fatigue. There was no relevant past medical history. The family history included a grandfather who died due to arachnoid hemorrhage. She reported a negative history for habits such as smoking and drinking. The history of presenting illness was as follows. In August 20XX, liver dysfunction was detected during a health examination, and the patient visited a nearby doctor. Computed tomography (CT) revealed multiple AV shunts in the liver, and the patient was placed under observation. In March 20XX + 3, she developed back pain, and CT performed at an emergency visit to a nearby hospital revealed evidence of intrahepatic bile duct dilatation. She was judged to require further investigation and was referred to our hospital's gastroenterology department in May 20XX + 3.

The patient (height: 154 cm; weight: 48.0 kg) presented with a body temperature of 36.2℃, blood pressure of 107/50 mmHg, pulse rate of 70 beats/min, respiratory rate of 17 breaths/min, and clear consciousness. Palpebral conjunctival anemia and yellowing of the bulbar conjunctiva were absent. The abdomen appeared flat/soft on physical examination. The liver was palpable with two fingers placed transversely below the right hypochondriac region. The spleen was not palpable. The patient was not taking any oral medication at presentation.

The findings of blood tests performed at first visit to another hospital (Table 1) were as follows. Increased ALP,γGTP and T-bil levels were observed, indicating cholestatic liver damage.

**Table 1:** Laboratory findings at first visit to another hospital (August 20XX)

Hematology		Blood chemistry	
WBC	4.20/μL	Total protein	6.4 g/dL
RBC	494 × 10^4^/μL	Albumin	3.7 g/dL
Hemoglobin	14.6 g/dL	BUN	13.2 mg/dL
Hematocrit	44.8%	Creatinine	0.71 mg/dL
PLT	20.0 × 10^4^/μL	T-Bil	2.75 mg/dL
		D-Bil	1.85 mg/dL
Coagulation		ALP	392 U/L(104–338)
PT	69.5%	LDH	274 U/L
INR	1.20	AST	37 U/L
		ALT	22 U/L
		γGTP	95 U/L(16–73)

*ALT* alanine transaminase; *AST* aspartate transaminase; *BUN* blood urea nitrogen; *D-Bil* direct bilirubin, *γGTP* gamma-glutamyl transpeptidase; *HCVAb* hepatitis C virus antibody; *HBsAg* hepatitis B surface antigen; *LDH* lactate dehydrogenase; *INR* international normalized ratio; *PLT* platelet; *PT* prothrombin time; *RBC* red blood cell; *T-Bil* total bilirubin; *WBC* white blood cell

The findings of blood tests performed at the time of referral (Table 2) were as follows. Elevated ALP and γGTP were observed, indicating cholestatic liver damage. The ALB value was lower than the data at the first visit to another hospital, and NH_3_ was also increased, suggesting a decline in liver reserve. Hepatitis virus markers were negative.

**Table 2:** Laboratory findings at referral (May 20XX + 3)

Hematology		Blood chemistry		Viral markers	
WBC	7.12/μL	Total protein	6.5 g/dL	HBsAg	(-)
RBC	415 × 10^4^/μL	Albumin	3.1 g/dL	HCVAb	(-)
Hemoglobin	13.0 g/dL	BUN	16 mg/dL	Immunity	
Hematocrit	40.2%	Creatinine	0.54 mg/dL	ANA	(-)
PLT	24.1 × 10^4^/μL	T-Bil	2.0 mg/dL	AMA2	(-)
Neutrophil	49.0%	D-Bil	0.9 mg/dL	IgG	1472 mg/dL
Lymphocyte	20.2%	ALP	336 U/L(38–113)	IgG4	42 mg/dL
Eosinophil	22.1%	LDH	247 U/L	IgM	164 mg/dL
Endocrine		AST	37 U/L	IgE	57 IU/mL
TSH	1.52 μIU/mL	ALT	20 U/L	CRP	2.373 mg/dL
fT4	1.3 ng/dL	γGTP	162 U/L(9–32)	Urine	
Coagulation		TC	156 mg/dL	Urinary sugar	(-)
PT	77.1%	BCAA/TYR NH3	2.53 66 µmol/L	Urinary protein	(-)
INR	1.12	BNP	184.5 ng/mL		

*ALT* alanine transaminase;* AMA2* anti-mitochondrial antibody 2; *ANA* antinuclear antibody; *AST* aspartate transaminase; *BCAA* branched chain amino acid; *BNP* brain natriuretic hormone; *BUN* blood urea nitrogen; *CRP* C-reactive protein; *D-Bil*direct bilirubin, *fT4* free tetraiodothyronine; γ*GTP* gamma-glutamyl transpeptidase; *HCVAb* hepatitis C virus antibody; *HBsAg* hepatitis B surface antigen; *LDH* lactate dehydrogenase; *Ig* immunoglobulin; *INR* international normalized ratio; *PLT* platelet; *PT* prothrombin time; *RBC* red blood cell; *T-Bil* total bilirubin; *TC* total cholesterol; *TSH* thyroid stimulating hormone; *TYR* tyrosine; *WBC* white blood cell

The findings of blood tests performed at the time of referral (Table 2) were as follows. Mild elevation in aspartate transaminase levels was observed. The albumin level was decreased, in addition to a significant reduction in the branched‐chain amino acid (BCAA)/tyrosine ratio to 2.53. The patient tested negative for the markers of viral hepatitis. Urinary proteins were absent.

**Fig. 1:**
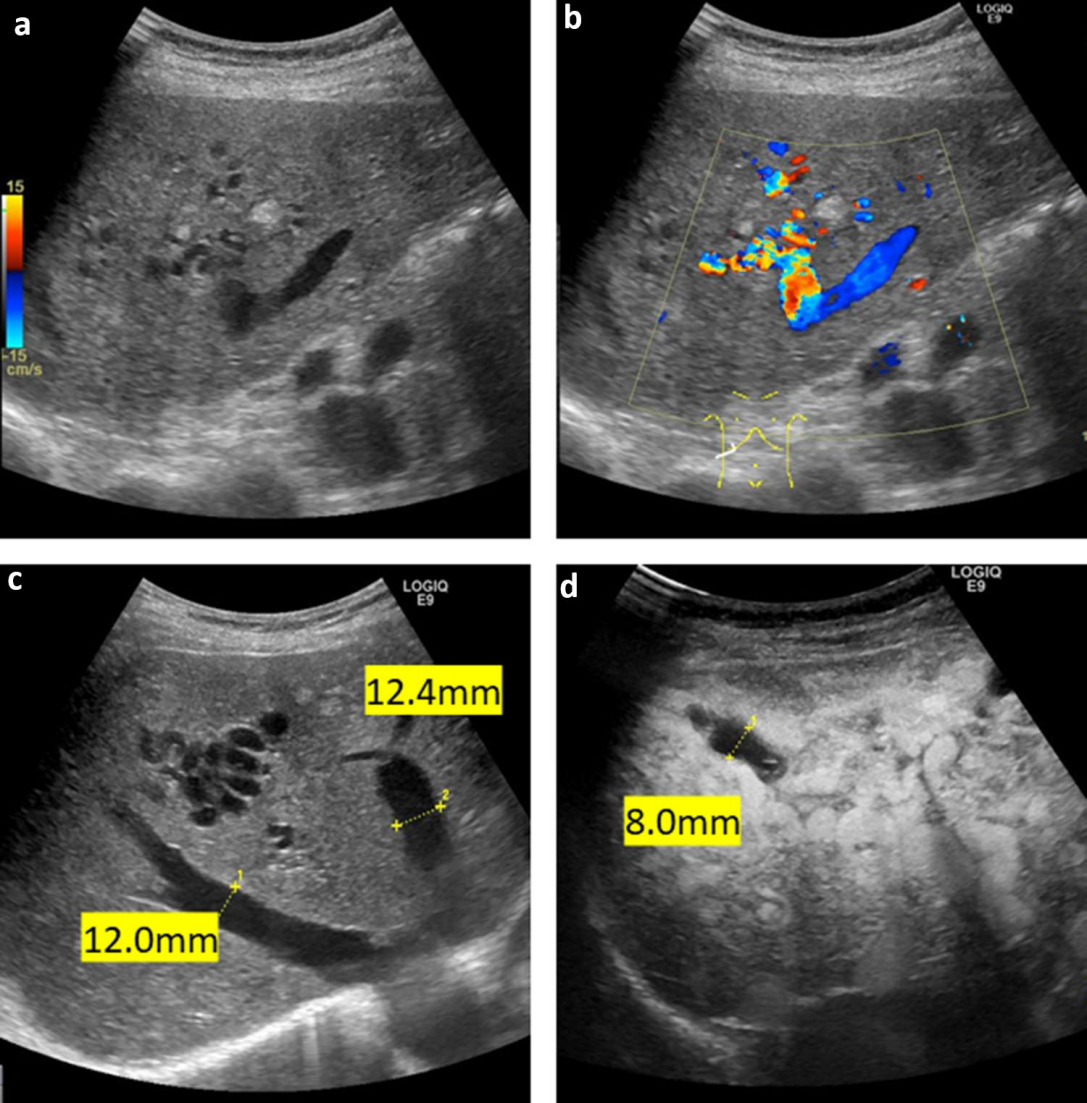
Abdominal ultrasonography (May 20XX + 3) **a** In the B-mode, arteriovenous anastomosis is observed in the right lobe of the liver. **b** Arteriovenous anastomoses are also observed on color Doppler imaging. **c**Dilated and tortuous arteries are observed. Moreover, the right hepatic vein (RHV) appears significantly expanded by 12 mm and the middle hepatic vein (MHV) by 12.4 mm. **d** Contrast-enhanced ultrasound shows a bile duct dilated to 8 mm in liver S6.

**Fig. 2:**
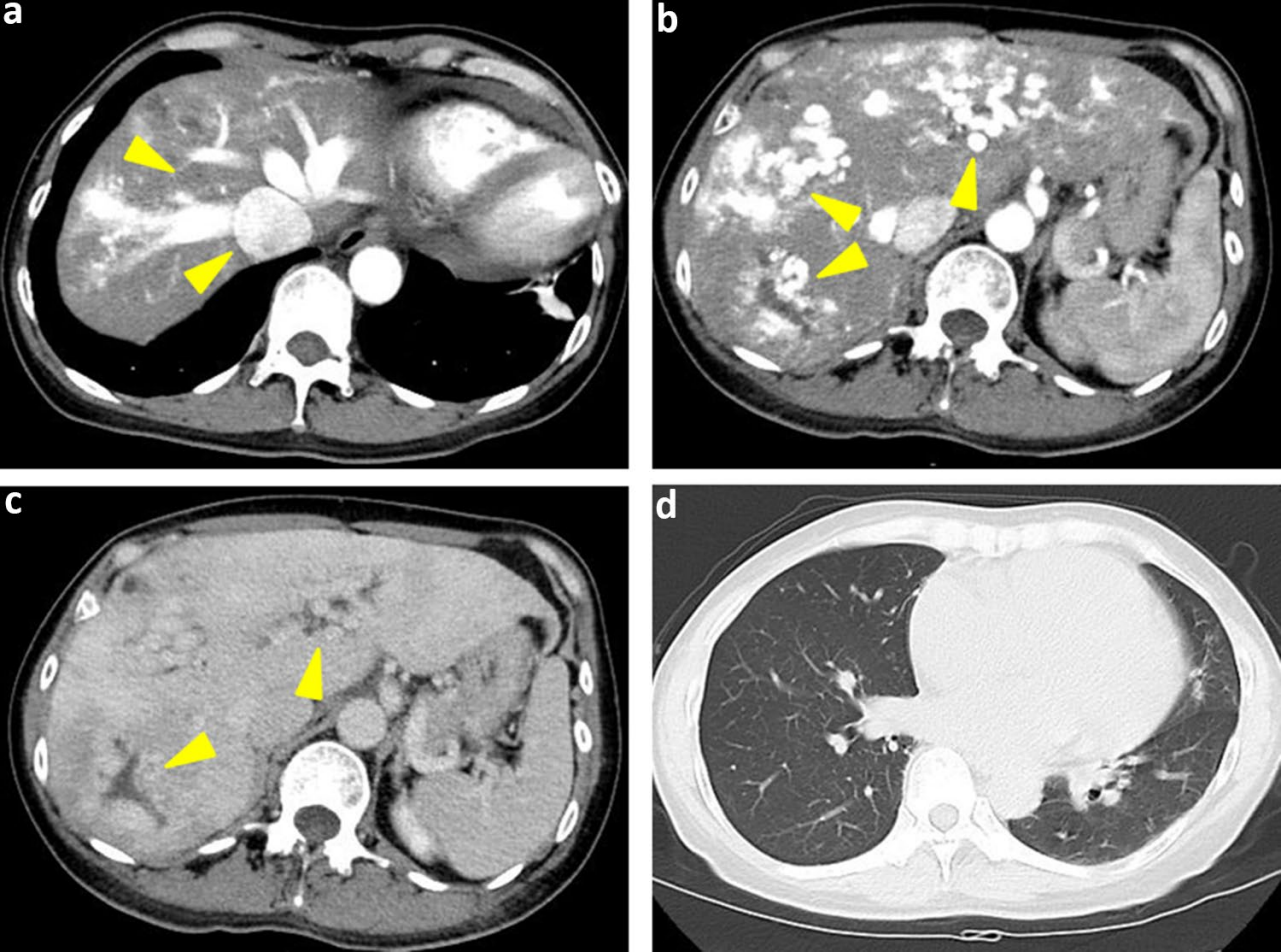
Contrast-enhanced computed tomography (June 20XX + 3) **a** Marked intrahepatic dilation of the hepatic vein (HV) and inferior vena cava (IVC) is observed during the arterial phase. **b** Dilated and tortuous arteries are observed in both lobes of the liver.** c** In the equilibrium phase, dilated bile ducts can be seen in both lobes of the liver. **d** No arteriovenous malformations were found in the lungs.

Abdominal B-mode ultrasonography revealed arteriovenous anastomosis in the right lobe of the liver (Fig. 1). Arteriovenous anastomoses were also observed on color Doppler imaging. Dilated and tortuous arteries were also observed. In addition, the right hepatic vein appeared significantly expanded by 12 mm and the middle hepatic vein by 12.4 mm. Contrast-enhanced ultrasound of the liver showed dilatation of the bile duct to 8 mm in S6. Contrast-enhanced CT examination (Fig. 2) revealed marked intrahepatic dilation of the hepatic vein (HV) and inferior vena cava (IVC), which was observed during the arterial phase. Dilated and tortuous arteries were observed in both lobes of the liver. In the equilibrium phase, dilated bile ducts were seen in both hepatic lobes. No AVMs were found in the lungs.

**Fig. 3:**
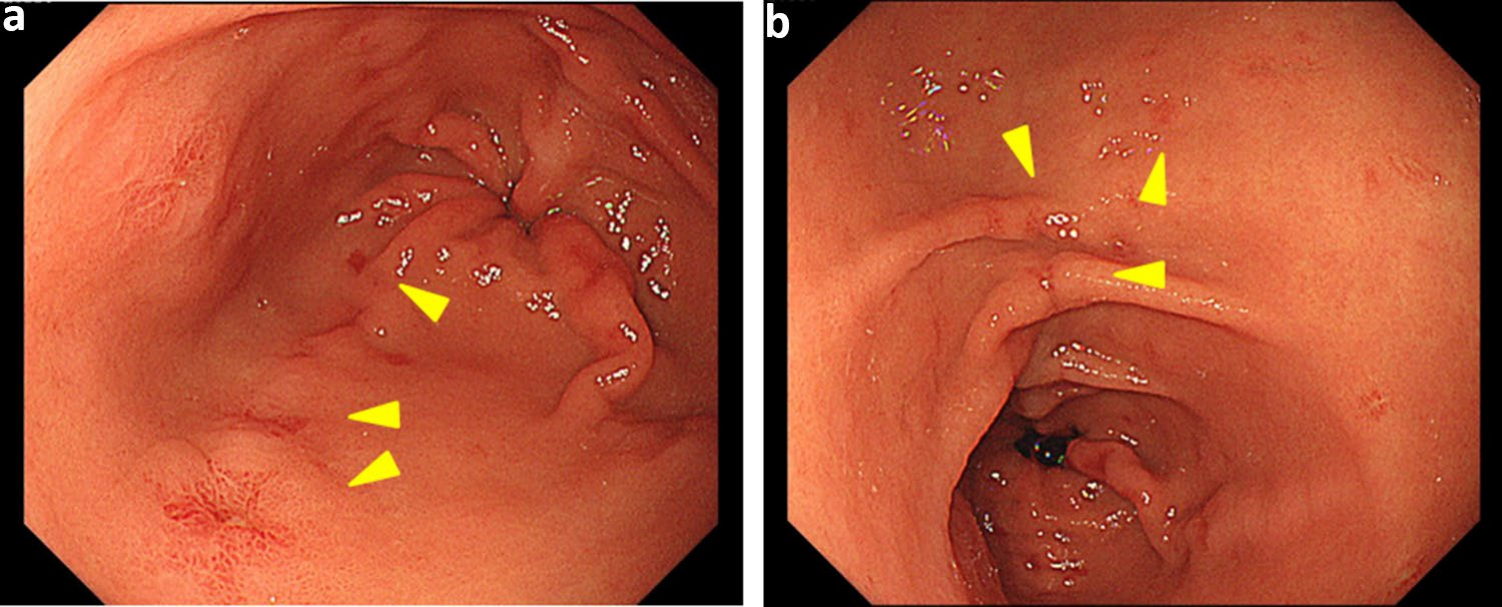
Upper endoscopy (May 20XX + 3) Scattered telangiectasia is evident mainly in the gastric antrum, in addition to the gastric mucosal findings associated with HHT.

**Fig. 4:**
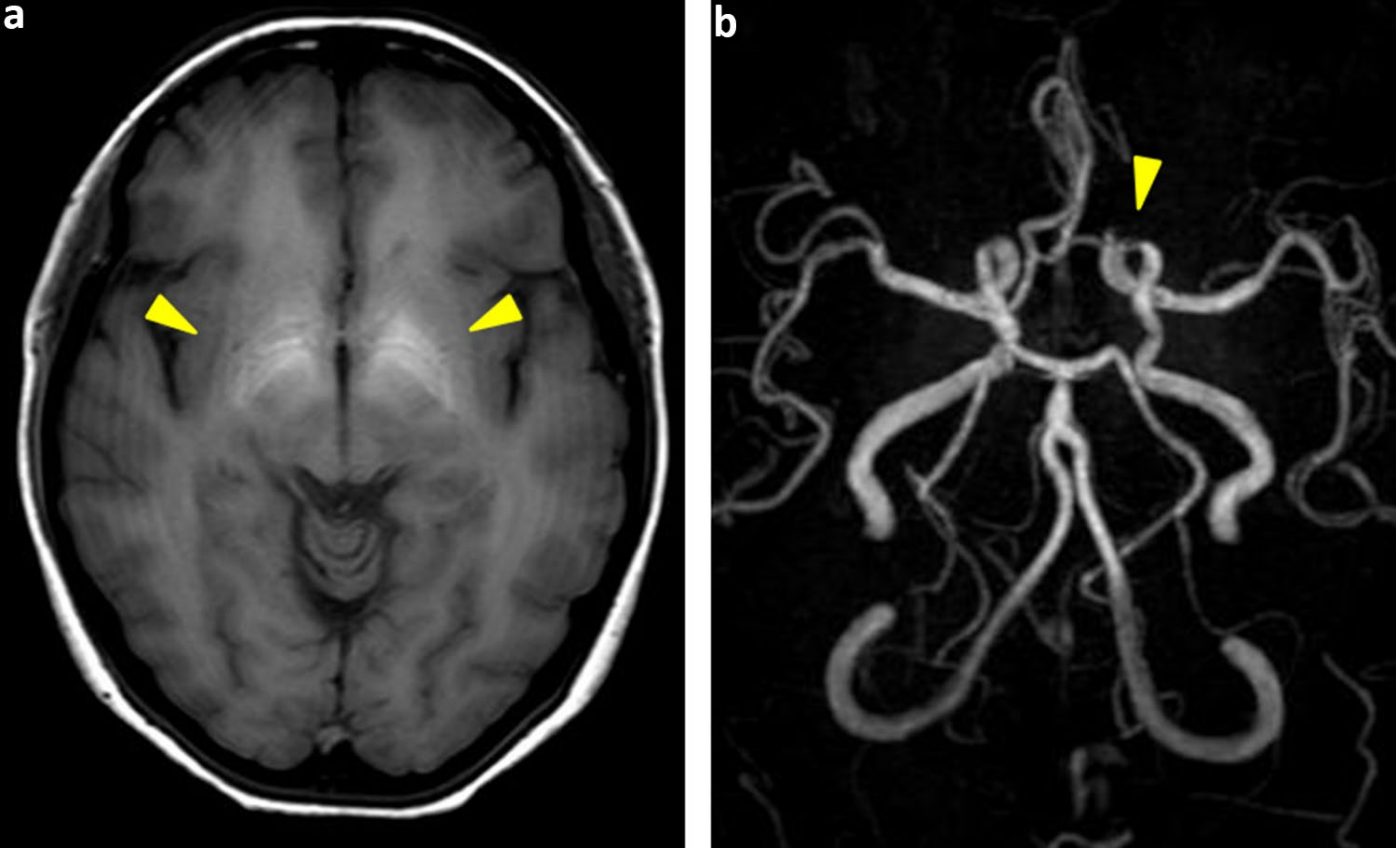
Head MRA examination (June 20XX + 3) **a** High-signal intensity is observed in the bilateral globus pallidus on T1-weighted imaging. **b** MRA shows decreased signal intensity in the left internal carotid artery (C3), suggesting the possibility of stenosis. MRA magnetic resonance angiography

Upper endoscopy (Fig. 3) revealed scattered telangiectasia, mainly in the gastric antrum; moreover, gastric mucosal findings associated with HHT were observed. High-signal intensity was observed in the bilateral globus pallidus on T1-weighted imaging. Head magnetic resonance angiography (MRA) (Fig. 4) revealed decreased signal intensity in the left internal carotid artery (C3), suggesting the possibility of stenosis.

Cardiac ultrasonography revealed that the right ventricular end-diastolic diameter was 39 mm (normal value 38 mm), indicating mild enlargement. The IVC diameter during expiration was 24 mm and that during inspiration was 16 mm; thus, the respiratory variation was reduced to less than 50%. Moderate tricuspid regurgitation (2.8 m/s) was observed, and the estimated right ventricular pressure was elevated to 46 mmHg (normal value 30 mmHg). The left ventricular ejection fraction was maintained at 65%. Cardiac catheterization showed that the mean pulmonary arterial pressure was mildly elevated at 25 mmHg (normal value less than 25 mmHg), and the pulmonary artery wedge pressure was 13 mmHg (values less than 15 mmHg indicate pulmonary arterial hypertension); thus, the patient was diagnosed with pulmonary arterial hypertension. Cardiac output was markedly elevated to 11.1, and the cardiac index was also elevated to 6.0 (normal value 2.2–4). This led to a diagnosis of hypercardiac pulmonary arterial hypertension. The obstructive hepatic venous pressure was 12 mmHg (normal value 11 mmHg), with no increase observed.

### Genetic testing

The patient underwent genetic testing for 4 genes related to Osler's disease (ENG, ACVRL1, SMAD4, BMPR2) using peripheral blood sampling. As a result, 2 variants were detected. The first, NM_001204.7 BMPR2 c.165 T > C p.Asn55 = , was a silent mutation, and the allele frequency in gnomAD was 0.000033, with a benign rating on ClinVar.

The other variant was NM_000118.3 ENG c.1429-9_1629-6dupATGGG, whose ClinVar evaluation was “conflicting interpretations of pathogenicity.” The allele frequency in gnomAD was 0.000092. No clear pathogenic variants were detected.

### Genetic counseling

Genetic counseling was performed before genetic testing while waiting for the results of genetic testing, and after these results were disclosed. Before the test, we explained the immutability, commonality, predictability, ambiguity, and ease of genetic testing. Since the patient was concerned about the effect of the above-mentioned mutations on her blood relatives, she seemed to be interested in genetic testing and consultation for her children. The patient’s eldest and fourth daughters were also present when the results were disclosed. We explained the conflicting interpretations of pathogenicity, the disease overview of HHT, and the possibility of updating variant evaluation.

By providing an opportunity to consult with the blood relatives before the test, we were able to share the test results with them.

### Clinical course after referral

HHT with hepatic AV shunt was suspected based on the results of contrast-enhanced CT performed at a nearby hospital, and additional tests were performed. Upper endoscopy performed in May 20XX + 3 depicted scattered telangiectasia mainly in the gastric antrum as well as gastric mucosal findings associated with HHT. Head MRA was performed in June 20XX + 3, and high-signal intensity was observed in the bilateral globus pallidus on T1-weighted imaging. These findings are reflective of decreased hepatic manganese clearance due to portal vein-HV shunts, consistent with HHT. Since the hepatic AV shunt was significant, echocardiography was performed in June 20XX + 3, revealing elevation in the estimated right ventricular pressure. Furthermore, right heart catheterization was performed in August 20XX + 3, and the mean pulmonary arterial pressure was found to be high at 25 mmHg, and the pulmonary artery wedge pressure was decreased to 13 mmHg. Furthermore, since the cardiac output value was as high as 11.1, a diagnosis of hypercardiac pulmonary arterial hypertension associated with a hepatic AV shunt was made. Administration of macitentan 10 mg/day, an endothelin receptor antagonist, was initiated for pulmonary hypertension.

## Discussion

Hepatic vascular malformations are observed in 3–31% of patients with HHT [11, 12]. Recent advances in diagnostic imaging have reportedly detected hepatic vascular malformations in 74% of HHT cases [13]. These hepatic vascular malformations are a frequent occurrence in HHT2 and rare in HHT1 [14]. Vascular lesions show diffuse distribution and range from telangiectasia without shunts to large vascular malformations with shunts. Hepatic vascular malformations include hepatic artery-portal vein shunts, hepatic artery-HV shunts, and portal vein-HV shunts, with the hepatic artery-portal vein shunt being the most common, although a combination of these may also occur [15, 16]. The clinical characteristics, such as hypercardiac right heart failure, portal hypertension, hepatic encephalopathy, and biliary ischemia due to the “steal” phenomenon, depend on the type of shunt [15]. In this patient, contrast-enhanced abdominal CT revealed that the hepatic artery-HV shunt was the primary cause, which was associated with high cardiac output and pulmonary arterial hypertension. Furthermore, multiple intrahepatic bile duct dilatation was present due to biliary tract ischemia arising from the hepatic artery “steal” phenomenon. In addition, T1-weighted head MRI showed high-signal intensity in the bilateral globus pallidus, suggesting manganese deposition. Since 98% of manganese absorbed from the gastrointestinal tract is excreted in the bile, manganese deposition in the globus pallidus primarily reflects the portal vein-HV shunt [15]. Since the portal vein-HV shunt is slightly difficult to visualize even on contrast-enhanced CT, manganese deposition in the globus pallidus constitutes supportive evidence of portal vein-HV shunting. Therefore, in this patient, a hepatic artery-HV shunt and portal vein-HV shunt were present. The serum albumin level was markedly decreased, along with a decrease in the BCAA/tyrosine ratio, which seems to reflect the decrease in the liver synthesis ability due to the portal vein-HV shunt. After referral to our hospital, the patient was prescribed a BCAA preparation. What is interesting about this case is that HHT was diagnosed as a result of liver damage. Hepatic vascular malformations are observed in approximately 30–70% of cases of HHT, but there are no reports of liver damage being the trigger for the diagnosis of HHT. Therefore, it seems important to suspect HHT as a differential diagnosis of liver damage for early diagnosis of HHT. In this case, hepatic artery-HVshunts and portal vein-HVshunts were present throughout the liver, and it is highly likely that ischemia to hepatocytes and bile duct cells was the cause of liver damage. Pulmonary arterial hypertension is a rare complication of HHT, whose diagnosis is based on the elevation of the mean pulmonary artery pressure to 20 mm Hg or higher on right heart catheterization. Revuz et al. reported that the frequency of pulmonary arterial hypertension in HHT is 4%, but the actual prevalence is thought to be much higher due to low screening rates [8, 9]. Pulmonary hypertension associated with HHT includes pulmonary arterial hypertension, which causes stenosis due to remodeling of the pulmonary blood vessels, and pulmonary hypertension due to hypercardiacity caused by the arteriovenous shunts. Pulmonary hypertension caused by a hepatic AV shunt is said to be a common occurrence in HHT [10]. In this case, right heart catheterization revealed a mildly elevated mean pulmonary artery pressure of 25 mmHg (normal value < 20 mmHg), and pulmonary artery wedge pressure of 13 mmHg (a value of less than 15 mmHg is denoted as pulmonary arterial hypertension), leading to the diagnosis of pulmonary arterial hypertension. In addition, the cardiac output was markedly elevated to 11.1, and the cardiac index was also elevated to 6.0 (normal value 2.2–4), resulting in the diagnosis of hypercardiac pulmonary arterial hypertension. Since pulmonary hypertension is caused by a hepatic AV shunt, transarterial embolization was our choice of treatment; however, this patient also had a portal vein-HV shunt. Hepatic artery embolization is a contraindication if a portal vein-HV shunt is present, owing to the high risk of serious complications such as postoperative hepatobiliary necrosis and death [15, 17, 18]. Therefore, simple hepatic artery embolization was not considered in this case. If the signs of liver failure or worsening of pulmonary hypertension become evident in the future, liver transplantation will be considered. Liver transplantation for HHT patients has been reported overseas. Eighteen (45%) of the 40 HHT patients had pulmonary hypertension. The 5-year survival rate after liver transplantation was 82.5%, demonstrating the effectiveness of liver transplantation. In addition, postoperative cardiac function was evaluated in 24 cases, and 18 cases (75%) reported improvement, and 5 cases (21%) showed no change [19]. However, in Japan, liver transplantation for HHT is not covered by insurance, which poses a hurdle to treatment.

In conclusion, we encountered a case of HHT with a hepatic AV shunt that was diagnosed as a result of liver damage. It is important to consider HHT caused by an intrahepatic shunt in the differential diagnosis of liver damage. This case report highlights the importance of a thorough systemic examination of the lungs, brain, gastrointestinal tract, and spine.
